# Supplementary material for: Monotonicity of fitness landscapes and mutation rate control
Source: J Math Biol. 2016 Apr 12;73(6):1491–524. doi: 10.1007/s00285-016-0995-3 (PMC5061859; doi:10.1007/s00285-016-0995-3)
Supplement: Supplementary file 1 — Supplementary material 1 (pdf 403 KB) [file 285_2016_995_MOESM1_ESM.pdf]

D Supplementary figures

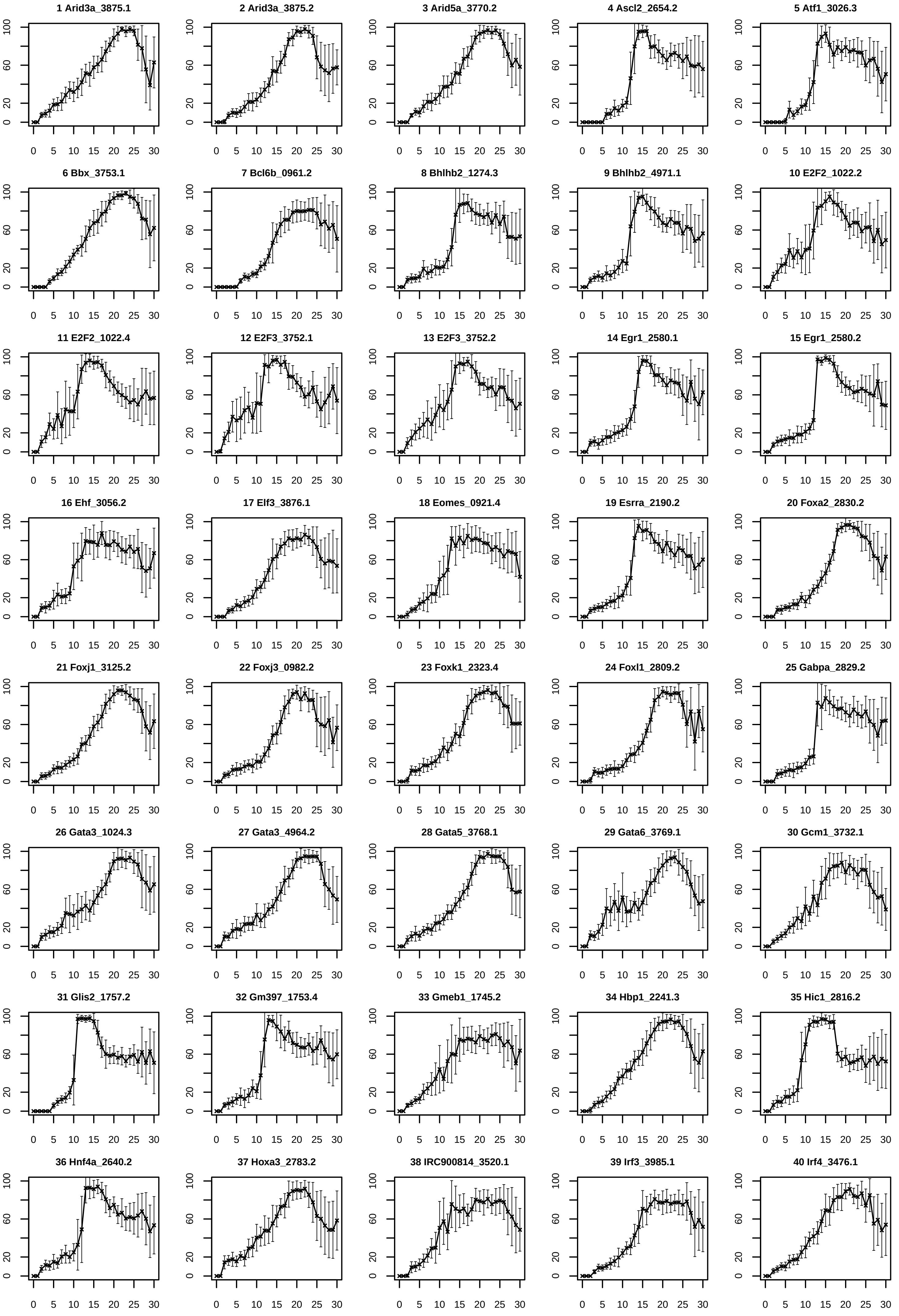
1 Arid3a_3875.1
2 Arid3a_3875.2
3 Arid5a_3770.2
4 Ascl2_2654.2
5 Atf1_3026.3
6 Bbx_3753.1
7 Bcl6b_0961.2
8 Bhlhb2_1274.3
9 Bhlhb2_4971.1
10 E2F2_1022.2
11 E2F2_1022.4
12 E2F3_3752.1
13 E2F3_3752.2
14 Egr1_2580.1
15 Egr1_2580.2
16 Ehf_3056.2
17 Elf3_3876.1
18 Eomes_0921.4
19 Esrra_2190.2
20 Foxa2_2830.2
21 Foxj1_3125.2
22 Foxj3_0982.2
23 Foxk1_2323.4
24 Foxl1_2809.2
25 Gabpa_2829.2
26 Gata3_1024.3
27 Gata3_4964.2
28 Gata5_3768.1
29 Gata6_3769.1
30 Gcm1_3732.1
31 Glis2_1757.2
32 Gm397_1753.4
33 Gmeb1_1745.2
34 Hbp1_2241.3
35 Hic1_2816.2
36 Hnf4a_2640.2
37 Hoxa3_2783.2
38 IRC900814_3520.1
39 Irf3_3985.1
40 Irf4_3476.1

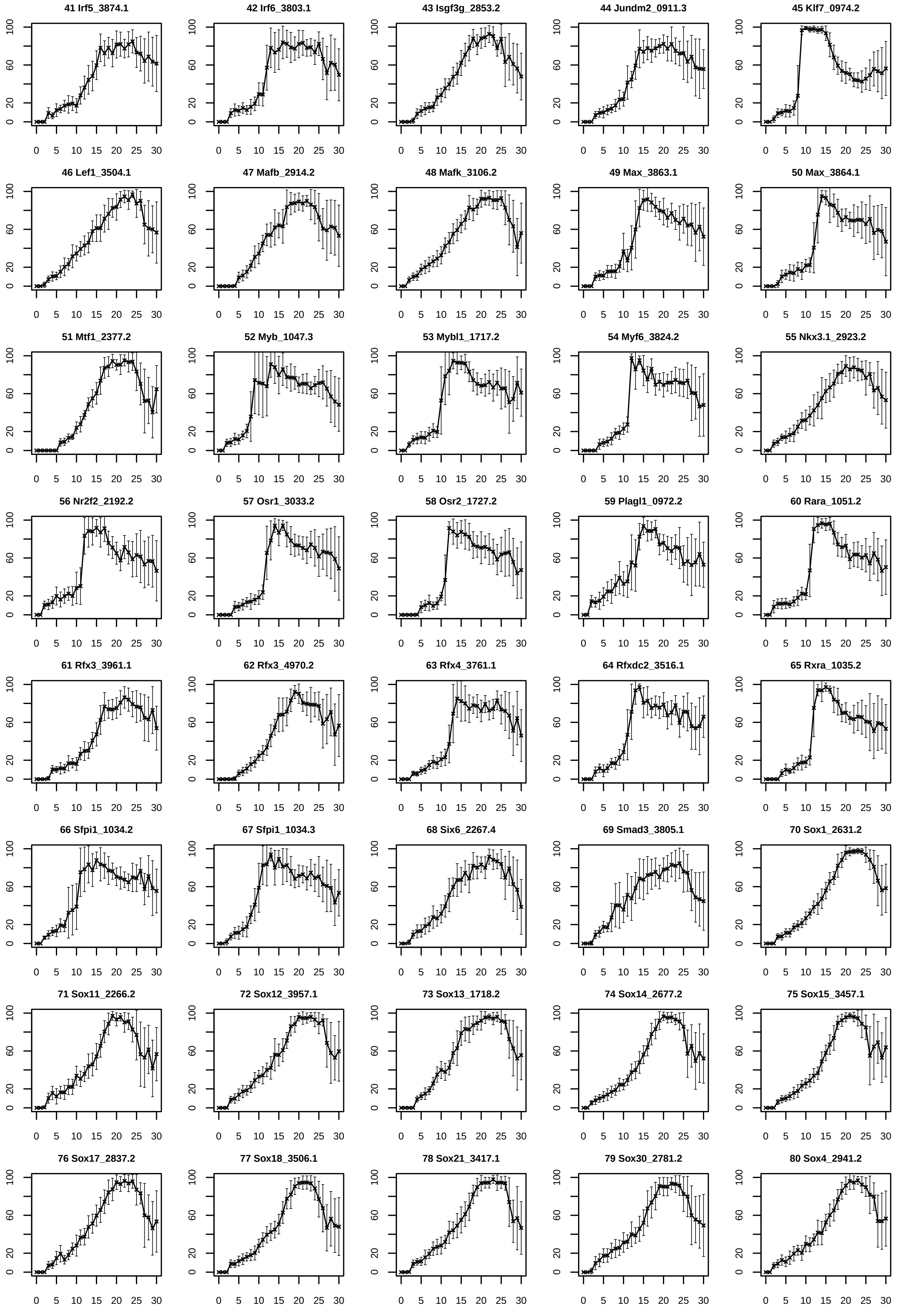
41 Irf5_3874.1
42 Irf6_3803.1
43 Isgf3g_2853.2
44 Jundm2_0911.3
45 Klf7_0974.2
46 Lef1_3504.1
47 Mafb_2914.2
48 Mafk_3106.2
49 Max_3863.1
50 Max_3864.1
51 Mtf1_2377.2
52 Myb_1047.3
53 Mybl1_1717.2
54 Myf6_3824.2
55 Nkx3.1_2923.2
56 Nr2f2_2192.2
57 Osr1_3033.2
58 Osr2_1727.2
59 Plagl1_0972.2
60 Rara_1051.2
61 Rfx3_3961.1
62 Rfx3_4970.2
63 Rfx4_3761.1
64 Rfxdc2_3516.1
65 Rxra_1035.2
66 Sfpi1_1034.2
67 Sfpi1_1034.3
68 Six6_2267.4
69 Smad3_3805.1
70 Sox1_2631.2
71 Sox11_2266.2
72 Sox12_3957.1
73 Sox13_1718.2
74 Sox14_2677.2
75 Sox15_3457.1
76 Sox17_2837.2
77 Sox18_3506.1
78 Sox21_3417.1
79 Sox30_2781.2
80 Sox4_2941.2

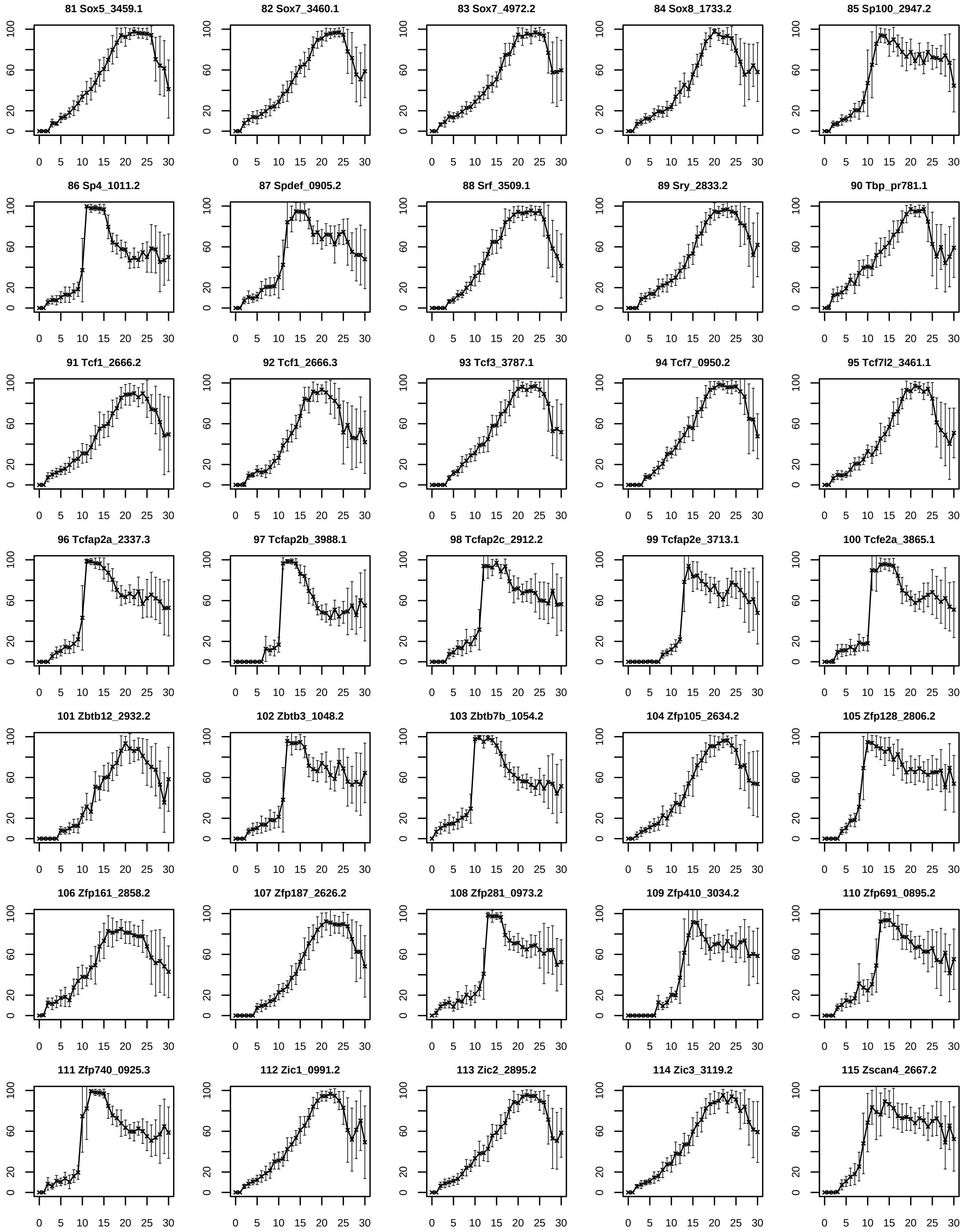
81 Sox5_3459.1
82 Sox7_3460.1
83 Sox7_4972.2
84 Sox8_1733.2
85 Sp100_2947.2
86 Sp4_1011.2
87 Spdef_0905.2
88 Srf_3509.1
89 Sry_2833.2
90 Tbp_pr781.1
91 Tcf1_2666.2
92 Tcf1_2666.3
93 Tcf3_3787.1
94 Tcf7_0950.2
95 Tcf7l2_3461.1
96 Tcfap2a_2337.3
97 Tcfap2b_3988.1
98 Tcfap2c_2912.2
99 Tcfap2e_3713.1
100 Tcfe2a_3865.1
101 Zbtb12_2932.2
102 Zbtb3_1048.2
103 Zbtb7b_1054.2
104 Zfp105_2634.2
105 Zfp128_2806.2
106 Zfp161_2858.2
107 Zfp187_2626.2
108 Zfp281_0973.2
109 Zfp410_3034.2
110 Zfp691_0895.2
111 Zfp740_0925.3
112 Zic1_0991.2
113 Zic2_2895.2
114 Zic3_3119.2
115 Zscan4_2667.2

Fig. 9 Optimal mutation rate control functions evolved by a Meta-GA on the transcription factor DNA-binding landscapes from (Badis et al, 2009). Ordinates show mutation rates, and abscissae show the binding scores. Each panel corresponds to a different transcription factor. Lines connect the average mutation rates obtained in 16 independent trials on a particular landscape. Errorbars represent standard deviations from the mean. The GAs do not spend much time at low binding scores meaning that the results become more random.

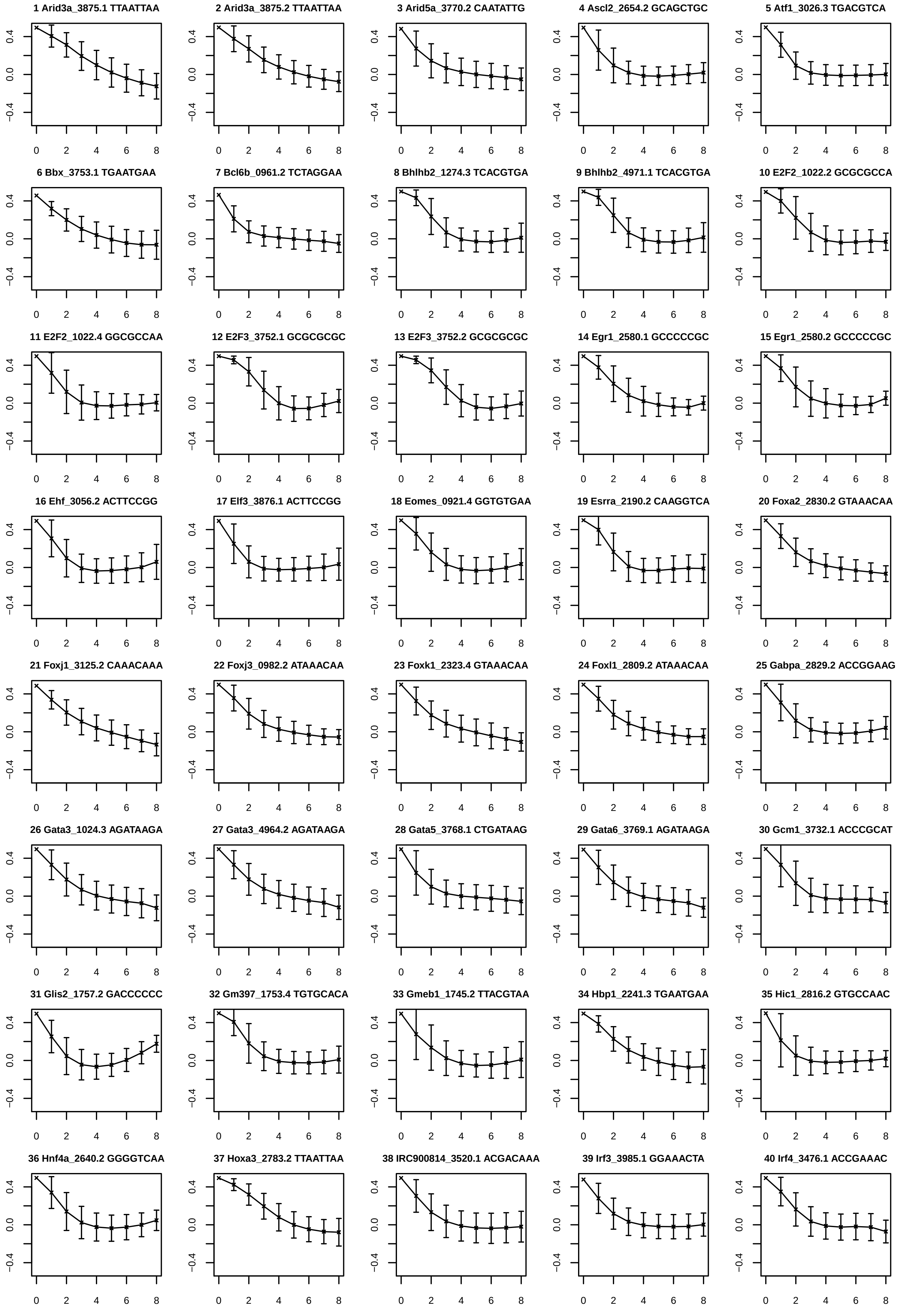
1 Arid3a_3875.1 TTAATTAA
2 Arid3a_3875.2 TTAATTAA
3 Arid5a_3770.2 CAATATTG
4 Ascl2_2654.2 GCAGCTGC
5 Atf1_3026.3 TGACGTCA
6 Bbx_3753.1 TGAATGAA
7 Bcl6b_0961.2 TCTAGGAA
8 Bhlhb2_1274.3 TCACGTGA
9 Bhlhb2_4971.1 TCACGTGA
10 E2F2_1022.2 GCGCGCCA
11 E2F2_1022.4 GGCGCCAA
12 E2F3_3752.1 GCGCGCGC
13 E2F3_3752.2 GCGCGCGC
14 Egr1_2580.1 GCCCCCGC
15 Egr1_2580.2 GCCCCCGC
16 Ehf_3056.2 ACTTCCGG
17 Elf3_3876.1 ACTTCCGG
18 Eomes_0921.4 GGTGTGAA
19 Esrra_2190.2 CAAGGTCA
20 Foxa2_2830.2 GTAAACAA
21 Foxj1_3125.2 CAAACAAA
22 Foxj3_0982.2 ATAAACAA
23 Foxk1_2323.4 GTAAACAA
24 Foxl1_2809.2 ATAAACAA
25 Gabpa_2829.2 ACCGGAAG
26 Gata3_1024.3 AGATAAGA
27 Gata3_4964.2 AGATAAGA
28 Gata5_3768.1 CTGATAAG
29 Gata6_3769.1 AGATAAGA
30 Gcm1_3732.1 ACCCGCAT
31 Glis2_1757.2 GACCCCCC
32 Gm397_1753.4 TGTGCACA
33 Gmeb1_1745.2 TTACGTAA
34 Hbp1_2241.3 TGAATGAA
35 Hic1_2816.2 GTGCCAAC
36 Hnf4a_2640.2 GGGGTCAA
37 Hoxa3_2783.2 TTAATTAA
38 IRC900814_3520.1 ACGACAAA
39 Irf3_3985.1 GGAAACTA
40 Irf4_3476.1 ACCGAAAC

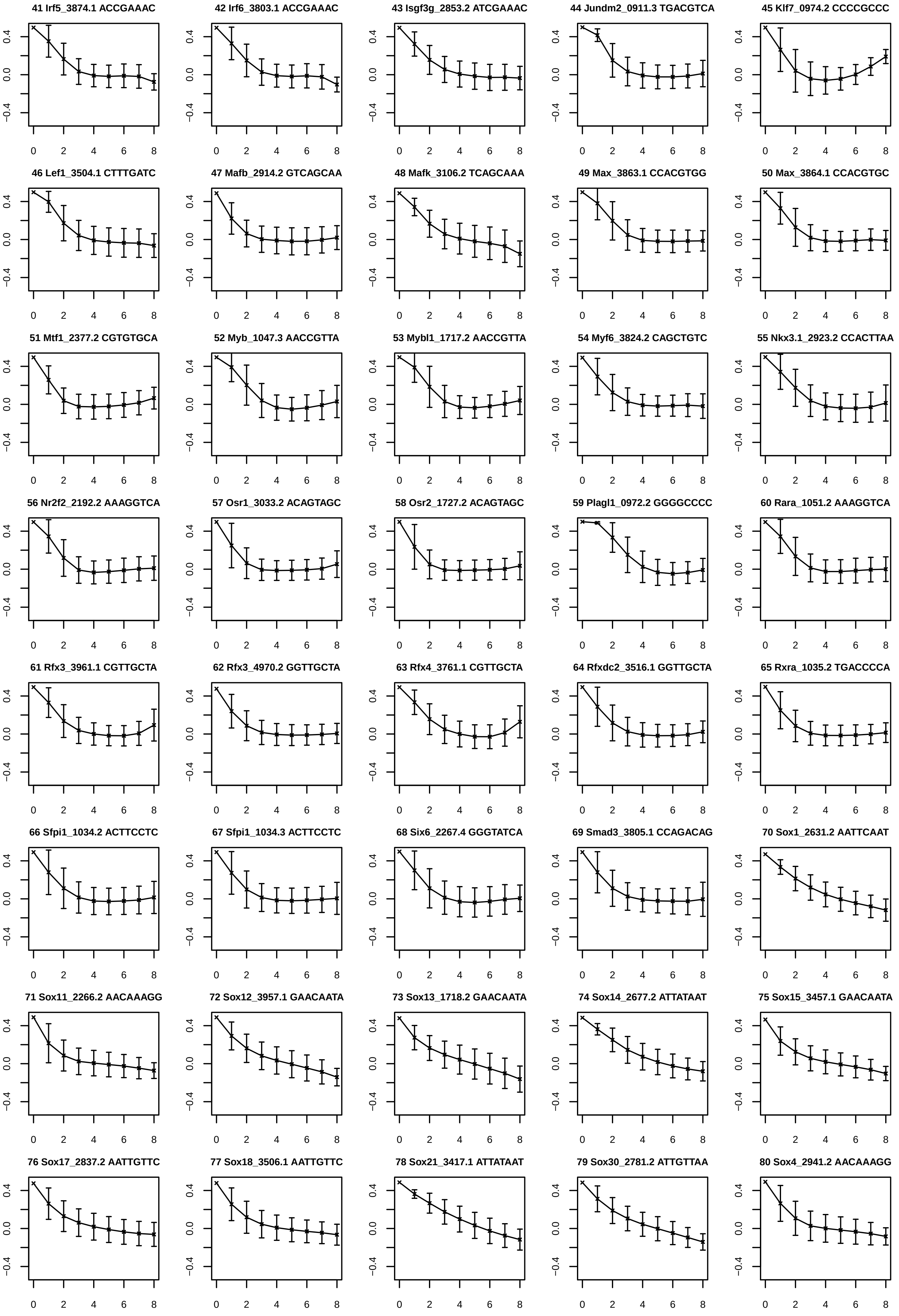
41 Irf5_3874.1 ACCGAAAC
42 Irf6_3803.1 ACCGAAAC
43 Isgf3g_2853.2 ATCGAAAC
44 Jundm2_0911.3 TGACGTCA
45 Klf7_0974.2 CCCCGCCC
46 Lef1_3504.1 CTTTGATC
47 Mafb_2914.2 GTCAGCAA
48 Mafk_3106.2 TCAGCAAA
49 Max_3863.1 CCACGTGG
50 Max_3864.1 CCACGTGC
51 Mtf1_2377.2 CGTGTGCA
52 Myb_1047.3 AACCGTTA
53 Mybl1_1717.2 AACCGTTA
54 Myf6_3824.2 CAGCTGTC
55 Nkx3.1_2923.2 CCACTTAA
56 Nr2f2_2192.2 AAAGGTCA
57 Osr1_3033.2 ACAGTAGC
58 Osr2_1727.2 ACAGTAGC
59 Plagl1_0972.2 GGGGCCCC
60 Rara_1051.2 AAAGGTCA
61 Rfx3_3961.1 CGTTGCTA
62 Rfx3_4970.2 GGTTGCTA
63 Rfx4_3761.1 CGTTGCTA
64 Rfxdc2_3516.1 GGTTGCTA
65 Rxra_1035.2 TGACCCCA
66 Sfpi1_1034.2 ACTTCCTC
67 Sfpi1_1034.3 ACTTCCTC
68 Six6_2267.4 GGGTATCA
69 Smad3_3805.1 CCAGACAG
70 Sox1_2631.2 AATTCAAT
71 Sox11_2266.2 AACAAAGG
72 Sox12_3957.1 GAACAATA
73 Sox13_1718.2 GAACAATA
74 Sox14_2677.2 ATTATAAT
75 Sox15_3457.1 GAACAATA
76 Sox17_2837.2 AATTGTTC
77 Sox18_3506.1 AATTGTTC
78 Sox21_3417.1 ATTATAAT
79 Sox30_2781.2 ATTGTTAA
80 Sox4_2941.2 AACAAAGG

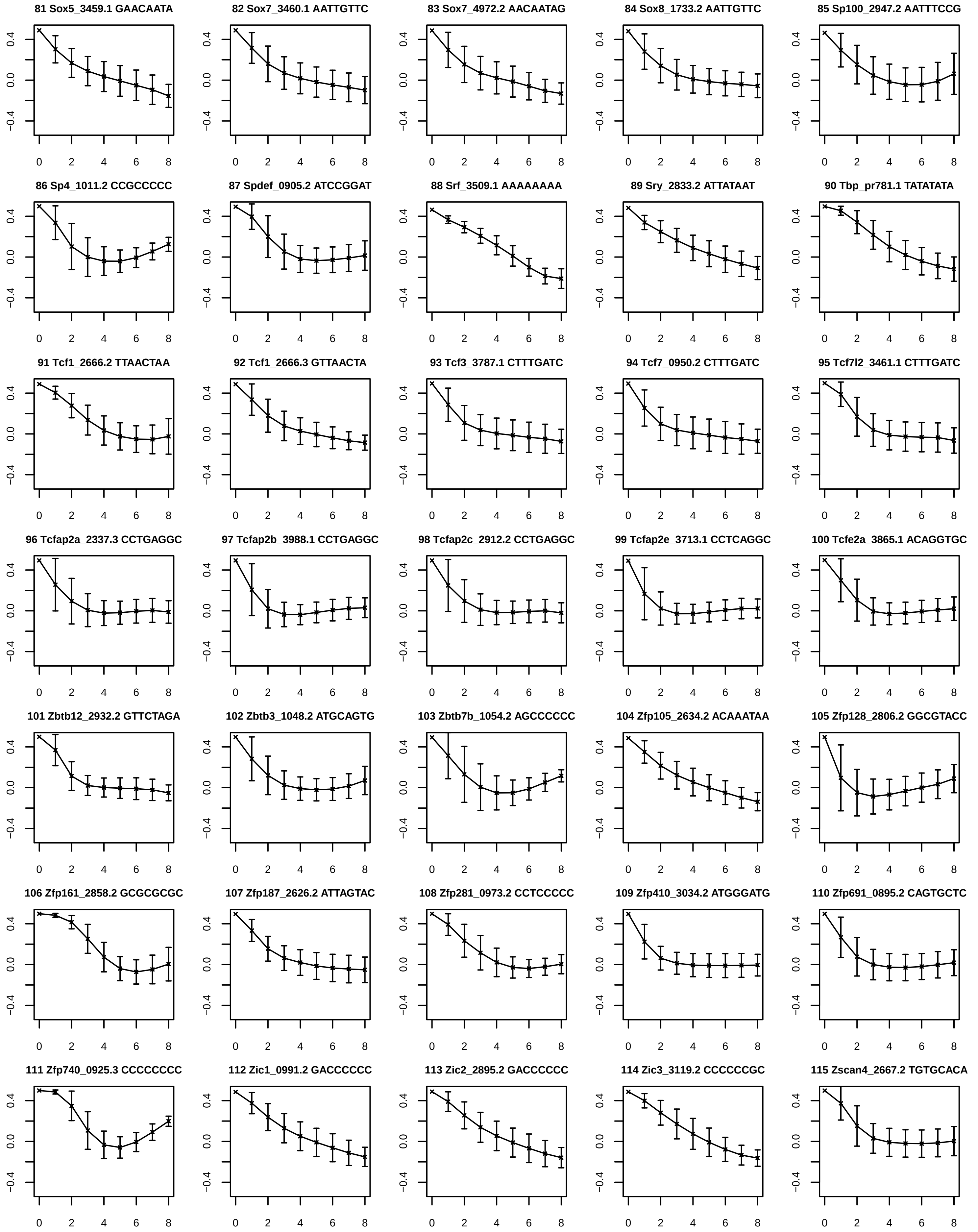
81 Sox5_3459.1 GAACAATA
82 Sox7_3460.1 AATTGTTC
83 Sox7_4972.2 AACAATAG
84 Sox8_1733.2 AATTGTTC
85 Sp100_2947.2 AATTTCCG
86 Sp4_1011.2 CCGCCCCC
87 Spdef_0905.2 ATCCGGAT
88 Srf_3509.1 AAAAAAAA
89 Sry_2833.2 ATTATAAT
90 Tbp_pr781.1 TATATATA
91 Tcf1_2666.2 TTAACTAA
92 Tcf1_2666.3 GTTAACTA
93 Tcf3_3787.1 CTTTGATC
94 Tcf7_0950.2 CTTTGATC
95 Tcf7l2_3461.1 CTTTGATC
96 Tcfap2a_2337.3 CCTGAGGC
97 Tcfap2b_3988.1 CCTGAGGC
98 Tcfap2c_2912.2 CCTGAGGC
99 Tcfap2e_3713.1 CCTCAGGC
100 Tcfe2a_3865.1 ACAGGTGC
101 Zbtb12_2932.2 GTTCTAGA
102 Zbtb3_1048.2 ATGCAGTG
103 Zbtb7b_1054.2 AGCCCCCC
104 Zfp105_2634.2 ACAAATAA
105 Zfp128_2806.2 GGCGTACC
106 Zfp161_2858.2 GCGCGCGC
107 Zfp187_2626.2 ATTAGTAC
108 Zfp281_0973.2 CCTCCCCC
109 Zfp410_3034.2 ATGGGATG
110 Zfp691_0895.2 CAGTGCTC
111 Zfp740_0925.3 CCCCCCCC
112 Zic1_0991.2 GACCCCCC
113 Zic2_2895.2 GACCCCCC
114 Zic3_3119.2 CCCCCCGC
115 Zscan4_2667.2 TGTGCACA

Fig. 10 Landscapes of binding score between 8 base-pair DNA sequences and transcription factors (TF) from (Badis et al, 2009). Ordinates show binding scores, and abscissae show Hamming distances from the top string (a sequence with the highest DNA-TF binding score). Each panel corresponds to a different transcription factor. Lines connect mean values of the binding score for each value of the Hamming distance from the top string. Errorbars represent standard deviations. Note that this dataset does not distinguish between sequences on opposite strands of the DNA. Therefore, a sequence and its reverse complement are shown only once and the Hamming distance shown is either to the top string or its reverse complement, whichever is the closer.
